# Gut microbiomes-host immune response and organ dysfunction across the gut-lung, gut-brain, and gut-liver axes

**DOI:** 10.3389/fcimb.2026.1918183

**Published:** 2026-09-07

**Authors:** Karthikeyan Sundaram, Venkataraman Prabhu, Harish Selvaraj

**Affiliations:** 1 Department of Herbal Pharmacology and Environmental Sustainability, Chettinad Hospital and Research Institute, Chettinad Academy of Research and Education, Kelambakkam, Chennai, Tamil Nadu, India; 2 Division of Medical Research, SRM Medical College Hospital and Research Centre, Kattankulathur, Chennai, Tamil Nadu, India

**Keywords:** dysbiosis, gut microbes, gut-brain axis, probiotics, SCFA

## Abstract

Microbial dysbiosis is associated with the onset and progression of disease and may influence host organ function through alterations in microbial metabolites, epithelial integrity, and immune signaling The gut communicates with distant organs through interconnected gut–lung, gut–brain, and gut–liver axes that contribute to physiological homeostasis. In the gut, short-chain fatty acid (SCFA)-producing microbes enhance mucin synthesis, epithelial barrier integrity, and immune regulation, while reduced intestinal pH may limit pathogen colonisation. In this review, we provide an integrated synthesis of evidence across the gut–lung, gut–brain, and gut–liver axes, focusing on how gut microbial dysbiosis and microbiota-derived metabolites influence host immune regulation and distant-organ function. Within the gut–brain axis, microbial metabolites, including SCFAs, can influence enteroendocrine signaling and vagal communication, thereby contributing to metabolic, emotional, and cognitive processes. Within the gut–liver axis, microbial alterations and metabolites are associated with hepatic metabolic dysfunction, inflammation, and lipid metabolic changes, with experimental evidence suggesting potential protective effects of *Akkermansia muciniphila.* By comparing the three gut–organ axes, this review identifies shared mechanisms involving SCFAs, microbial metabolites, epithelial barrier integrity, immune signaling, and neuroendocrine communication, while highlighting the current evidence gaps and priorities for future mechanistic and translational research.

## 1 Introduction

The gut is a crucial organ that plays an essential role in regulating the body’s overall mechanisms (Shibi Anilkumar et al., 2025; Mallick et al., 2024). The gut microbiome influences distant organs, particularly the brain, liver, and lungs, through direct and indirect interactions involving microorganisms and their metabolites. Among these interconnected communication networks, this review focuses primarily on the gut–lung, gut–brain, and gut–liver axes (Jeyanathan et al., 2022). In the gut-lung axis, microbial dysbiosis is associated with the development and progression of conditions such as asthma, chronic obstructive pulmonary disease (COPD), cystic fibrosis, and bronchiectasis (Narayana et al., 2023). However, identifying the role of the intestinal microbiota in various lung disorders has resulted in the formulation of the gut-lung axis concept, and digestive issues have also been observed in respiratory illnesses (Alharris et al., 2022).

The gut microbiota comprises various bacteria, viruses, fungi, archaea, and other microorganisms. In particular, dysbiosis of this microbiota population leads to multiple infections and affects mental health and behavior. Predominantly, the phyla of microbiota in mammalian guts include *Bacteroidetes, Firmicutes, Proteobacteria, Actinobacteria, Verrucomicrobia*, and *Fusobacteria* (Eribo et al., 2020). In particular, the most common gut microbial metabolites are short-chain fatty acids (SCFAs), which increase mucin production and lower intestinal pH. These mechanisms protect epithelial integrity, boost the immune system, and prevent pathogens from sticking (Sun et al., 2024; Jardou and Lawson, 2021).

SCFAs regulate immune cell function through G protein-coupled receptor (GPCR) signaling and epigenetic mechanisms involving histone deacetylase (HDAC) inhibition. SCFAs increase histone acetylations and alter the transcription of immune regulatory genes; SCFA-mediated HDAC inhibition can regulate the differentiation of naïve T cells into effector and regulatory subsets through modulation of the mTOR-S6K pathway (Park et al., 2015). Butyrate has been shown to suppress inflammatory CD4^+^ T-cell activity and reduce the production of IFN-γ and IL-17, while under appropriate TGF-β1 conditions it can promote Foxp3^+^ regulatory T-cell differentiation and contribute to intestinal immune homeostasis. Additionally, SCFA can control innate lymphoid cell responses. Butyrate can modulate the epithelial barrier function and mucosal immunological homeostasis by stimulating IL-22 production by ILCs and CD4^+^ T cells through pathways involving GPR41 and HDAC inhibition. However, the effects of SCFA-mediated immune modulation depend on the cellular context, the surrounding cytokine milieu, and SCFA concentration. Therefore, HDAC inhibition offers a crucial epigenetic mechanism by which SCFAs produced from the gut microbiota can convert microbial metabolic activity into modifications in the phenotype of host immune cells and inflammatory reactions (Wang et al., 2025; Sankarganesh et al., 2025).

Additionally, gut γδ T cells are linked to sepsis-induced lung injury. Recent research suggests that CCL1, produced by alveolar macrophages and regulated by the Wnt signaling pathway in early sepsis, may play a crucial role in coordinating the migration of small intestinal γδ T17 cells to the lung parenchyma. This is supported by the strong chemotactic effect of pulmonary CCL1 on these cells (Xie et al., 2024). In this review, we provide an integrated synthesis of evidence across the interconnected gut–lung, gut–brain, and gut–liver axes, focusing on how gut microbial dysbiosis and microbiota-derived metabolites influence host immune regulation and distant-organ function.Within the gut–lung axis, we discuss associations with respiratory disorders, including asthma, chronic obstructive pulmonary disease (COPD), cystic fibrosis, bronchiectasis, pulmonary hypertension, and lung cancer. The gut–brain axis is considered in relation to cognitive impairment, neuroinflammation, mood and behavioral changes, neurodegenerative disorders, and Parkinson’s disease, whereas the gut–liver axis is discussed in relation to metabolic dysfunction-associated steatotic liver disease (MASLD), liver inflammation, fibrosis, oxidative stress, and ferroptosis. Particular emphasis is placed on microbial dysbiosis, short-chain fatty acids (SCFAs), microbial metabolites (Huang et al., 2020), epithelial barrier integrity, immune signaling, and neuroendocrine pathways as potential mechanisms linking gut microbial alterations with distant-organ outcomes. By comparing these gut–organ axes, this review identifies shared mechanisms involving microbial dysbiosis, SCFAs, microbial metabolites, epithelial barrier integrity, immune signaling, and neuroendocrine pathways, while highlighting differences in the available evidence and key gaps for future mechanistic and translational research.

## 2 Search methodology

This review examines gut and its association with different organs such as brain, lung and liver. Specifically, gut-lung axis, gut – brain axis, gut – liver axis, gut linked to immune response, and association with the dysfunctions of the multiple organs. The following keywords were used: gut- brain axis, gut – liver axis, gut – lung axis, functions of the gut, gut links to immune response, and gut links to various disorders. The study utilized various academic databases, including PubMed/Medline, Science Direct, Springer Link, Scopus, and the WHO report. A total of 35211 published studies in English were examined from 2020 to 2025, and 88 studies were selected to evaluate the gut and links to multiple organs and factors associated with the immune response, and various disorders. However, minireviews, duplicates, insufficient data, and outdated literature were excluded. A total of 7 studies were identified for analysis.

## 3 Gut-lung axis

Gut microbial alterations have been associated with respiratory disorders, including asthma and COPD, and may influence pulmonary outcomes through epithelial barrier and immune mechanisms (Ma et al., 2025). Interestingly, while several Haemophilus species were found in gut microbiota profiles. At the same time, gut microbiota profiles revealed a negative correlation between COPD and several Haemophilus species, while Streptococcus was linked to the beginning of asthma; exacerbations of COPD and respiratory failure have been associated with changes in the airway microbiome, namely Pseudomonas species. Especially Pseudomonas A and E, there was a substantial correlation between NCBI taxonomy taxa and asthma and COPD (Liu et al., 2023). Specifically, the epithelial barrier plays a vital role in preserving structural integrity and immunological equilibrium in the intestine and lungs. However, by forming a barrier with adherent junction proteins like E-cadherin and tight junction proteins like ZO-1 and Claudin-1, epithelial cells protect local tissues and reduce distant inflammatory damage. Inflammation undermines the integrity of epithelial barriers in the gut and lungs. Pro-inflammatory cytokines, including IFN-γ, TNF-α, and IL-1β, increase cell permeability by downregulating junction proteins. Impaired lung and gut barrier function results in disruption and dysfunction (Wang et al., 2024; Han et al., 2019; Sim et al., 2018). Notably, in children with cystic fibrosis, the common genus Akkermansia identified in stool specimens correlated negatively with calprotectin and positively with FEV1% pred. *Akkermansia* reduces inflammation, although its effects on lung function are unknown. However, it supports CF patients’ gut-lung axis and multi-system interaction (McKay et al., 2023).

Recently, a study investigated how lipopolysaccharide (LPS) induces acute lung injury (ALI). The study showed that LPS activates the TLR4/NF-κB signaling pathway, leading to increased production of the pro-inflammatory cytokines IL-1β, IL-6, and TNF-α.

Fecal microbiota transplantation (FMT) effectively restores a balanced gut microbiota composition by enhancing the population of beneficial commensal bacteria that produce SCFAs. This process inhibits TLR4/NF-κB activation, thereby reducing pro-inflammatory factors and oxidative stress (Druszczynska et al., 2024; Tang et al., 2021). Furthermore, gut microbiota affects ILC2 phenotype and function. Reduced intestinal acetate and butyrate levels were linked to a decrease in the abundance of SCFA-associated bacterial taxa in a mouse model of acute COPD exacerbation. Butyrate administration in experiments decreased lung inflammation and inflammatory ILC2 responses, indicating a possible function of microbiota-derived SCFAs in controlling gut–lung immunological communication. These results, however, should not be taken as proof that particular bacterial taxa directly cause changes in the human microbiome linked to COPD because they are mostly based on experimental models. Reductions in *Hungateiclostridiaceae* and *Clostridiaceae* have also been documented in smokers, underscoring the possible impact of smoking-related microbial changes on the gut–lung axis. Crucially, several potential confounding factors may affect the relationships between gut microbiota and lung diseases. Lung function and gut microbial composition can be separately impacted by smoking exposure, including duration and intensity of smoking. Age, food choices, medication use, and environmental exposures such biomass smoking and air pollution can also influence the composition of the microbiota and the risk of respiratory diseases. Future research should take these aspects into consideration when assessing potential causative links within the gut–lung axis, and detected changes in gut microbiota between patients with and without COPD should be interpreted cautiously (Jiang et al., 2023; Sepahi et al., 2021; Kirschner et al., 2021). Noticeably, the LPAL group exhibited a higher abundance of *Sporobacter*, an acetate-producing bacterium. However, in left pulmonary artery ligation-pulmonary hypertension (LPAL-PH), a reduction in beneficial SCFA-producing bacteria, such as *Eubacterium butyrate* and *Eubacteriaceae propionate*, may alter gut epithelial cells and enhance gut permeability (Chen et al., 2022; Kim et al., 2020). LPAL groups exhibited heightened gut permeability, demonstrating that butyrate mitigates inflammation and safeguards the gut barrier. Propionate markedly decreased heart hypertrophy, fibrosis, vascular dysfunction, and hypertension, thereby attenuating cardiovascular disease progression (Chen et al., 2022).

The gut microbiota metabolite acetate reduced inflammation via the FFAR2-AMPK pathway. Various studies suggest that the gut microbiota and metabolites regulate type I interferons (IFNs). Both adaptive and acquired immunity are affected by the gut microbiota. Comparable findings show that the gut microbiota impacts the production of CD4 and CD8 IAV-specific T cells and the antibody response after infection (Hu et al., 2024; Niu et al., 2023). Also, the gut microbiome significantly correlates with immune response; besides, the gut-lung link clarifies the pathway by which the lymphatic system might convey bacteria to the lungs. SCFAs produced or altered by gut microbiota may affect pulmonary function. Meanwhile, peptidoglycans from bacterial cell walls generate Nod1 ligands via lysozyme action, and circulating Nod1 ligands can reach the lungs or mature gut immune cells. Therefore, it influences early-life innate immune system characteristics and guides the adaptive immune system (Abdelgawad et al., 2023).

Gut bacteria associated with respiratory disorders have been found via metagenomic sequencing. However, a metagenomic sequencing investigation found that COPD patients had greater *Streptococcus* levels in their feces than healthy controls (Bowerman et al., 2020). Similarly, a study examining 88 potential associations found that increased levels of Streptococcus are linked to a higher risk of developing COPD. Air pollution and long-term smoking cause COPD, the third largest cause of mortality worldwide. It’s caused by inflammation and immune reactions (Zhou et al., 2024).

Notably, a recent Mendelian randomization (MR) study found a positive correlation between *Holdemanella* abundance and COPD risk but a negative correlation with family XIII in people with diabetespeople with diabetes with cognitive impairment. On the contrary, other studies have revealed an intriguing negative relationship between *Holdemanella* abundance and propionate levels, an SCFA (Cheng et al., 2024). *In vitro* studies showed that sodium butyrate (NaB) is an SCFA, and pretreatment inhibited lung invasive germs. NaB also significantly reduced lung inflammatory cytokines and MRSA-induced lung inflammation (Zhao Y et al., 2023). MR also evaluates disease-risk factor causality. Genetic variations are instrumental factors in MR. A recent study found that there are links between Eubacterium ruminantium and SCLC. Lung cancer (LC) and its subtypes were substantially associated with five gut microbe (GM) groups at the genome-wide statistical significance threshold. One group had LC, one NSCLC, one SCLC, two lung adenocarcinomas, and no lung squamous carcinoma. No heterogeneity or horizontal pleiotropy was found in the sensitivity analysis, which was reliable. Reverse MR analysis indicated reverse causation between GM abundance and genetic sensitivity to LC and its subtypes in three of 47 statistically significant GM groups (Ma et al., 2024).

On the other hand, the bacterium Allisonella may reduce COPD risk. *Allisonella* flora, a kind of gut bacteria, may create and metabolize histamine, inhibit intestinal microbial ectasia, and restore the intestinal mucosa (Niu et al., 2025). The MR investigation found an inverse causal connection between *Allisonella* and COPD, suggesting that *Allisonella* may lessen the risk of COPD (Wei et al., 2023). Besides this, a recent study revealed that Nlrp6-/- mice may have a diminished ability to fight lung infections, including Streptococcus pneumoniae infections that induce COPD exacerbations. NOD-like receptor family pyrin domain containing (NLRP) 6 reduces inflammation in response to *Staphylococcus aureus* or *S. pneumoniae* infections, which may benefit subsequent infections (Nascimento et al., 2023).

Gut microbes are intrinsically linked to host defense mechanisms against infections (**Table 1**), and they rely on IL-1β generated by innate immune cells in response to pathogens and damage patterns. NLRP3 activation by saturated fats like palmitic acid releases IL-1β, whereas inflammasome activity regulates its production. Probiotic bacteria such as *Bifidobacterium* and *Lactobacillus* can modulate IL-1β production, although the resulting effects depend on the bacterial strain, host immune status, and inflammatory context. During infection or tissue injury, excessive IL-1β production can promote inflammatory responses and contribute to tissue damage, whereas appropriately regulated IL-1β signaling is an important component of host defense. Besides that, *Lactobacillus paracasei MI29 enhanced IL-1β production in the lungs of IFNAR−/− mice but did not provide substantial protection against influenza virus infection (*Kim et al., 2023*). This finding indicates that increased IL-1β production alone does not necessarily translate into improved host protection and should be interpreted in the context of the broader inflammatory response.* (Kim et al., 2023). This finding indicates that increased IL-1β production alone does not necessarily translate into improved host protection and should be interpreted in the context of the broader inflammatory response.

**Table 1:** Gut- associated with various organs and immune responses.

Study	Study population (n)/clincial manifestation	Outcomes	Axis network	Diagnostic platform
Narayana et al., 2023	57/primary ciliary dyskinesia (9%), Immunodeficiency (13%), and postinfection (7%) were the most commonly found etiologies.	Streptococcus and Fusobacterium inhabited the lungs, while bacteriodes inhabited the gut. Most gut and lung fungus were Candida. Anatomical site-specific β-diversity investigations revealed significant ecological differences, with matched gut-lung specimens exhibiting lower bacterial diversity in the lung.	Gut-Lung	16s rRNA and 18s rRNA sequencing
Xie et al., 2024	Acute respiratory distress syndrome (ARDS) or acute lung injury (ALI) causes sepsis.	The primary source of IL-17A in the lung is increased pulmonary γδ T cells 24 hours post-CLP. S-KT decreased sepsis-induced intestinal barrier breakdown, but CLP increased intestinal permeability 24 hours later. S-KT significantly reduced MUC-2 expression and protein loss 24 hours after CLP.	Gut-Lung	study with Il-7rflox/flox/TrdcCreERT2, and male wild-type (WT) C57BL/6 J mice
Wang et al., 2023	Severe acute pancreatitis (SAP)- acute lung injury (ALI)	Severe acute pancreatitis (SAP) animals had a higher Firmicutes/Bacteroidetes ratio in their gut microbes. Simpson, Chao1, Shannon, and total species indices were lower than controls. SAP- acute lung injury (ALI) decreases gut bacterial diversity. Also, Acetate, propionate, butyrate, and valerate were much lower in the SAP-ALI group, and all groups had distinct SCFA counts.	Gut-Lung	Principle component analysis (PCA)
DuPont et al., 2023	Parkinson’s disease, fecal microbiota transplantation (FMT)	Lactobacillaceae, Limnochordaceae, and Peptostreptococcaceae were substantially and selectively more abundant in fecal microbiota transplantation (FMT) treatment than placebo after 13 weeks. In the placebo group, Proteobacteriaceae was more prevalent.	Gut-Brain	lyophilized FMT oral randomized, double-blind, placebo-controlled pilot study
Onimus et al., 2024	Long-term spatial reference memory. object and recognition/spatial (location) memory	animals’ T-maze ability to identify familiar from unfamiliar environments. Both experimental groups produced identical findings, proving recognition deficits do not need spatial/environmental dysfunctions.	Gut-brain vagal axis	Novel place recognition (NPR) test
Hai et al., 2025	glucose and tolerance, proinflammatory cytokines,	Il33 ΔIEC mice showed increased lipid consumption genes (*Ppara* and *Cpt1a*) and reduced lipid synthesis (Srebp2) and absorption (*Cd36*), Intestinal IL-33 deletion reduced fibrosis-related gene expression (*Acta2, Tgfb1*, and *Col1a1*) and proinflammatory cytokines (*Tnf, Il1b*, and *Il6*). HFD-fed Il33 ΔIEC animals showed lower blood ALT and AST levels.	Gut-Liver axis	animal study with Il33 ΔIEC mice, Il33 fl/fl controls
Lv et al., 2024	Acetaminophen/paracetamol (APAP) - induce acute lung injury (ALI)	Serum levels of the cytokines eotaxin, MIP-1α, IL-1β, IL-3, IL-12p70, IL-13, IL-17α, IFN-γ, and TNF-α were reduced in mice, G-CSF, MCP-1, CXCL-1, IL-1α, and IL-6 were increased. Lacticaseibacillus casei Shirota (LcS) counteracted the APAP-induced decrease in eotaxin and increased IL-1α levels.	Gut-Liver axis	Animal study with pathogen-free C57BL/6 mice

## 4 Gut-brain axis

Gut microbiota impact neurodegenerative illnesses via modulating gut-brain immunity; communication between the central nervous system and gastrointestinal tract is bidirectional (Lu et al., 2022). However, corticotropin releasing factor (CRF) is released by the central nervous system (CNS) and the intestines in response to stress. Both substances can activate CRF receptor 1 (CRFR1) by influencing the enteric nervous system (ENS) of the gut. This activation regulates various intestinal processes, including bacterial balance, immune and inflammatory responses, neuropeptide and hormone release, as well as intestinal secretion, sensation, and motility. This is referred to as the brain-gut axis (Xu et al., 2022).

Disruption of the gut-brain vagal axis impacted long-term recognition memories but not motor, procedural, reference, or working memory. Also, hippocampal markers including increased expression of immediate-early genes (*cFos, Arc, Npas4*, and *FosB*), altered dendritic spine shape, and imbalanced synaptic plasticity are linked to behavioral deficits. As substrates or functional proxies, maladaptive indicators preserve learning and memory. Stimulating the whole vagus nerve (cervical level) improves learning and memory in humans and rats (Onimus et al., 2024). It may boost hippocampus neurogenesis and plasticity. Initial studies confirmed this. However, specific gut-brain vagal manipulations (ablations) impact hippocampal activities, showing that the gut is a major peripheral organ that strongly influences learning and memory (Olsen et al., 2022; Sanders et al., 2019; Onimus et al., 2024). Also, metabolic products from gut microbes stimulate enteroendocrine cells to release neuropeptides and hormones, affecting appetite and mood via the vagus nerve. They also regulate hormone precursors, modulating stress and mood, and contributing to global immunity and brain function (Xiang et al., 2024).

The gut probiotic plays a crucial role in inducing anti-inflammatory effects and reducing pro-inflammatory effects. However, distinct alterations in certain bacterial groups at the genus level were observed: a decrease in the genus *Wolbachia* and an increase in the genera *Akkermansia*, *Acetobacter*, *Strenotrophomonas*, and *Lactobacillus*. Variations in abundance may result in oxidative stress and altered metabolites, subsequently decreasing amyloid plaque deposition, neuroinflammation, and neuronal injury. In animal models of ageing, *Lactobacillus* species have been shown to possess anti-oxidative properties (Siripaopradit et al., 2024). Consequently, the term psychobiotics refers to probiotic strains used to treat mental diseases and may improve host health. Many bacterial byproducts, including neuroactive metabolites and inflammatory mediators, can cross the gut and blood–brain barriers, allowing passage through the BGM system. In addition, calcitonin gene-related peptide (CGRP) modulates gut-brain axis activity. However, increased pathogenic microorganisms can disrupt the gut microbiota, causing neurons to produce CGRP. Hence, CGRP secretion during infection activates host defense and the calcitonin receptor (CRLR) immunological response. CGRP modulates the gut-brain axis, whereas probiotic microorganisms balance the intestinal microbiota. The HPA axis releases CRH and ACTH, which react to inflammation and can elicit immune responses against bacteria, viruses, and fungi (Oroojzadeh et al., 2022; Stefanakis et al., 2024; Wei et al., 2018). In addition, whether low-dose cannabis could treat Microbiota–gut–brain axis (MGBA) imbalance and how HIV/SIV infection may cause neuroinflammation and neuroimmune activation remains unclear. Delta-9-tetrahydrocannabinol (THC) can also override *miR-142-3p*, a neuroinflammatory miRNA that is increased in the Basal ganglia (BG) of vehicle-treated SIV (VEH/SIV) but not in THC/SIV Rhesus macaques. Chronic THC increased the relative abundance of SCFA producers *Clostridium butyricum, Faecalibacterium prausnitzii, Butyricicoccus pullicaecorum, Firmicutes, Clostridia, Lactobacilli*, and *Bifidobacteria* and IPA-producing *Clostridium botulinum, paraputrificum*, and *cadaveris*, which positively modulated the colonic microbiome (McDew-White et al., 2023; Keimpema et al., 2021).

Notably, the Brain-derived neurotrophic factor (BDNF) helps host neuronal cells mature and differentiate for peripheral nervous system (PNS) development (Camuso et al., 2022). Vagus nerves (VN) alter brain BDNF expression. Likewise, glucagon-like peptide-1 (GLP-1) and cholecystokinin impact hippocampus BDNF gene production via VN receptors to control hunger. VN-deficient mice have low hippocampal BDNF gene expression. In 4- and 6-week-old mice, Vago dramatically decreased nucleus of solitary tract (NTS) neuronal excitability and BDNF production and release in the hippocampus and paraventricular nucleus of the hypothalamus (PVH). Like Vago mice with typical microbiota transplants, they gained weight and ate more. These data imply VN regulates appetite and GI signals (Ge et al., 2024). The hippocampus and amygdala generated less BDNF in germ-free mice, limiting brain development. *Akkermansia muciniphila* increases BDNF gene expression in depression-model mice by altering gut microbiota and metabolites (Ding et al., 2021).

Deficiencies in distal colon hormone genes, such as *Gcg, Pyy*, and *Insl5*, may lead to metabolic disorders. Reduced 15-minute GLP-1 and insulin responses in EEC^ΔCol^ mice may contribute to the glucose intolerance observed in weight-matched mutant mice, akin to the findings in Gcg^ΔCol^ mice (Tan et al., 2024; Panaro et al., 2020). Similarly, colonic Gcg deficiency and global Insl5 deletion did not result in hyperphagia or obesity. EEC influences microbiota by modulating colonic motility and the feeding dynamics within the intestinal lumen. The enteric endocrine system and microbiota function in a reciprocal manner. Comparative analysis with another study indicates that microbial colonisation influences Insl5 and GLP-1 synthesis, while SCFA sensing via FFAR2, a SCFA receptor, enhances PYY production and the number of PYY-expressing cells (Brooks et al., 2016; Larraufie et al., 2018; Tan et al., 2024). In the gut-brain axis, the signal’s convergence into a single class of vagal neurons (VIP–UTS2b) shows the circuit’s basic logic: once gut cells are activated, the circuit merely needs gut–brain transmission to generate behavioural preference (Li et al., 2022; Li et al., 2025; Tan et al., 2020).

A recent study highlighted that each vitamin has gut receptors. Nutrient stimulus identity is unnecessary. A limited number of intestinal cholecystokinin (CCK)-positive enteroendocrine cells (EECs) express sugar (SGLT1), fat (GPR40 and GPR120), and an unknown amino acid receptor. CCK signals sugar and nutrient sensing in the gut. These gut tissue single-cell RNA atlases from humans and animals suggest this (Bai et al., 2022). Also, the colon had about 700 differentially expressed genes (DEGs), mostly associated with the olfactory transduction pathway, after transcriptome analysis with *Hemerocallis citrina* Baroni (Daylily, DHC). After combining transcriptomics and network pharmacology, seven targets overlapped: *Alb, Drd2, Igf2, Pon1, Tshr, Mc2r*, and *Nalcn*. Moreover, qPCR showed that DHC effectively reduces colon *Pon1*, *Alb*, and *Cnr1* expression. This novel integrated transcriptome and network pharmacology approach helps us comprehend DHC’s anti-constipation effects (Lana and Giovannini, 2023; Liang et al., 2023).

## 5 Gut-liver axis

Liver disease has been linked to gut microbial dysbiosis, intestinal endotoxemia, and bacterial translocation. Using 16S rRNA sequencing and LEfSe analysis (D’Mello et al., 2021; Martínez-Sanz et al., 2023). examined the makeup of gut microbiota in adults with MASLD, including those living with HIV. The study comprised 20 HIV-negative individuals with MASLD, 30 HIV-positive individuals without MASLD, and 30 HIV-positive individuals with MASLD. LEfSe analysis used an LDA score >4 and an adjusted p-value <0.05 in the Kruskal–Wallis test to identify genera that contributed to group differences. *Ruminococcus, Streptococcus, Holdemanella, Blautia*, and *Lactobacillus* were more abundant in those with MASLD, while *Prevotella, Bacteroides, Dialister, Acidaminococcus, Alloprevotella*, and *Catenibacterium* were more prevalent in those without MASLD. These findings indicate that MASLD was associated with distinct gut microbial patterns; however, the study was observational and therefore does not establish a causal relationship between these microbial differences and MASLD development or progression (Martínez-Sanz et al., 2023).

An imbalance between reactive oxygen species and antioxidant defenses contributes to hepatic oxidative stress and may promote the progression of MASLD. Gut microbial dysbiosis can influence liver disease through several interconnected metabolic pathways. For example, alterations in bacteria carrying choline TMA-lyase (CutC/D) can increase conversion of dietary choline to trimethylamine (TMA), which is subsequently oxidized by hepatic flavin-containing monooxygenases to form trimethylamine N-oxide (TMAO). Recent experimental evidence indicates that intestinal IL-33 promotes the abundance of TMAO-producing bacteria and increases microbiota-derived TMAO, whereas reducing IL-33 or inhibiting TMAO synthesis attenuates metabolic dysfunction, hepatic oxidative stress, inflammation, and fibrosis in MASLD models (Hai et al., 2025).

Bile acid metabolism and signaling may potentially be affected by gut microbial dysbiosis. While other microbial enzymes help convert primary bile acids into secondary bile acids, gut bacteria express enzymes including bile salt hydrolases that deconjugate primary bile acids. These microbiota-mediated modifications can alter the composition of the intestinal bile acid pool and may also affect host receptors, specifically the Takeda G protein-coupled bile acid receptor 5 (TGR5) and the farnesoid X receptor (FXR). Dysregulated FXR/TGR5 signaling may affect bile acid production, enterohepatic circulation, hepatic lipid and glucose metabolism, and inflammatory reactions. Therefore, changes in the gut microbiota may impact liver disease through a linked route that includes altered bile acid composition and signaling, changes in microbial enzyme activity and metabolite production, and consequent metabolic and inflammatory disorders in the liver (Ridlon et al., 2014). This microbiota–bile acid signaling system may be further influenced by host genetic diversity. Major bile acid-sensing receptors include FXR (encoded by NR1H4) and TGR5 (encoded by GPBAR1). Genetic variations altering these receptors may alter bile acid responsiveness and subsequent metabolic or inflammatory signaling. The significance of host receptor signaling in influencing the effects of microbiota-derived compounds is further supported by experimental investigations showing that modified FXR or TGR5 activity can affect gut microbial composition and liver phenotypes (Xiang et al., 2026). However, there is still little concrete evidence connecting certain NR1H4 or GPBAR1 mutations to microbiota-dependent bile acid sensing and liver illness, indicating a crucial field for further research.

Lipopolysaccharide (LPS), the primary component of endotoxins, can enhance liver inflammation and accelerate the progression of liver fibrosis by activating intracellular signal transduction through the liver’s toll-like receptor 4 (TLR4), a phenomenon known as the “gut-liver” axis (Xu et al., 2022). Gut epithelial cells in the small and large intestines also produce large numbers of intestinal-type fatty-acid-binding proteins (I-FABP). Promoting fat uptake and metabolism. However, the patients with MASLD have greater I-FABP levels than healthy people. Healthy people had lower LPS levels than the MASLD group. Unlike I-FABP and LPS, Occludin positively linked with BMI without hepatic steatosis. Obesity promotes intestinal permeability (Dumitru et al., 2025).

The gut microbiota of the IgM-negative group, *B. fragilis* and *B. catenulatum* were selectively enriched, reducing immunological activation and serological indications of illness. Polysaccharide A (PSA), a cell wall component, induces Tregs and upregulates IL-10, modulating the immune system. These data support the taxon’s enrichment in the IgM-negative group, which had reduced liver damage (Karandikar et al., 2023). Interestingly, a stool bacterium *Akkermansia muciniphila* stimulates SCFA and treats metabolic disorders. Especially, *A. muciniphila* reduced liver peroxidation and normalized transaminase, alanine aminotransferase (ALT), aspartate aminotransferase (AST) and peroxide accumulation, since ALT and AST used as common indicator for liver injury (Rao et al., 2021).

**Figure 1:**
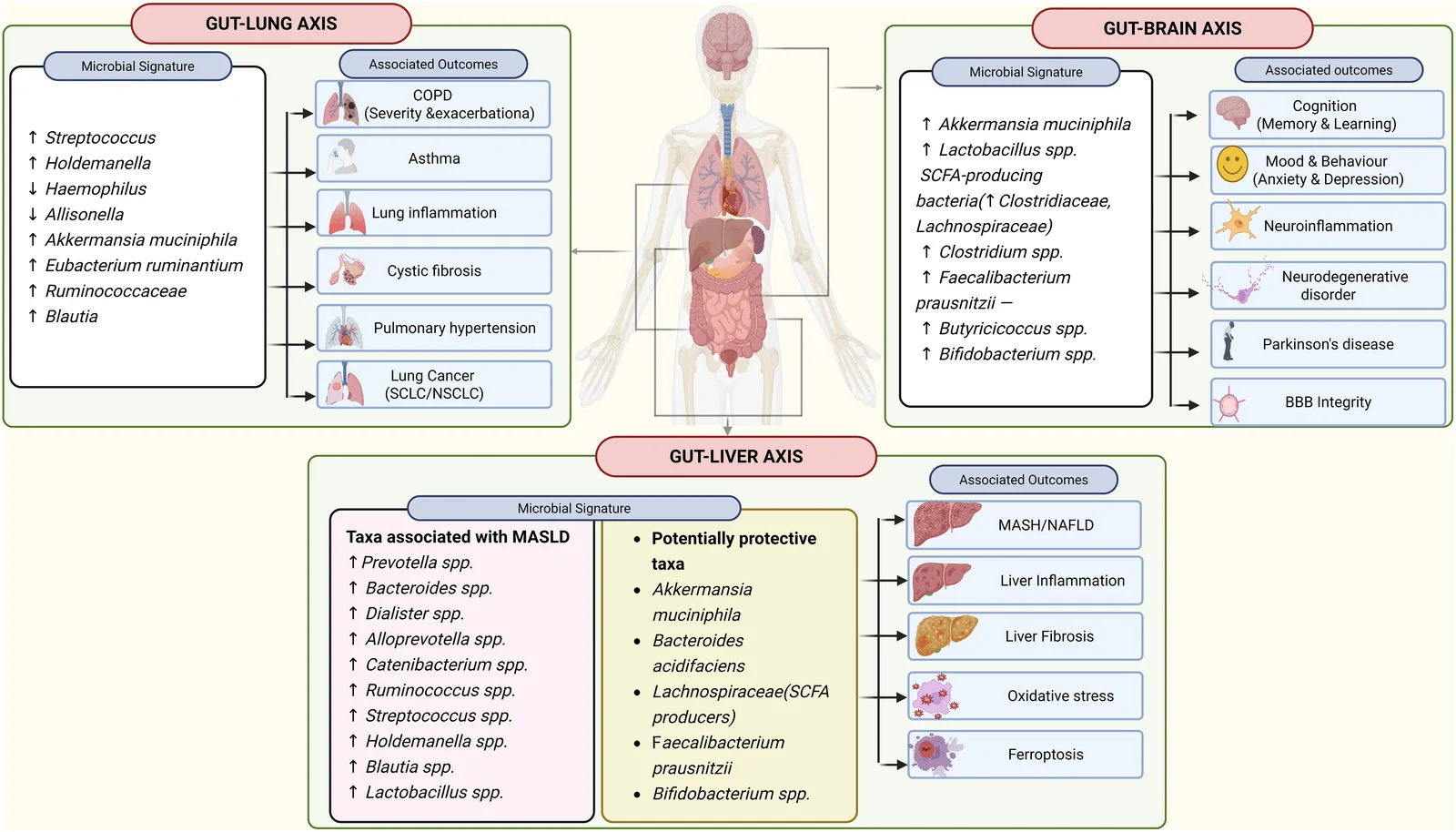
Gut microbial signatures and associated outcomes across the gut–lung, gut–brain, and gut–liver axes. The figure summarizes microbial taxa associated with respiratory, neurological, and hepatic outcomes. The gut–lung axis includes microbial changes associated with COPD, asthma, cystic fibrosis, pulmonary hypertension, and lung cancer. The gut–brain axis highlights taxa associated with cognition, mood, neuroinflammation, neurodegenerative disorders, Parkinson’s disease, and blood–brain barrier integrity. The gut–liver axis shows taxa associated with MASLD, liver inflammation, fibrosis, oxidative stress, and ferroptosis, including potentially protective taxa. The microbial changes shown primarily represent reported associations and should not be interpreted as evidence of causality unless supported by genetic or experimental studies. Host factors such as diet, medication, age, and environmental exposure may also influence these associations. Overall, the figure highlights shared and axis-specific relationships between gut microbial composition and distant-organ health and disease.

The hepatic lipidomic results indicated that *A. muciniphilia* therapy lowered free arachidonic acid (ARA) and ARA-PEs, whereas high fat- high fructose diet (HHFFD) feeding increased them. Due to oxidative stresses, arachidonic acid (ARA)-anchored PEs oxidize and damage the cell membrane, causing ferroptosis (Zhuge et al., 2025). Iron accumulation and lipid peroxidation are defining characteristics of ferroptosis, a distinct kind of non-apoptotic programmed cell death. Inhibition of ferroptosis has shown promise in mitigating hepatic ischemia-reperfusion injury (HIRI), non-alcoholic fatty liver disease, and alcoholic liver disease (ALD). Noticeably, the Lachnospiraceae bacteria trigger the KEAP1-NRF2 pathway, a crucial antioxidant signaling mechanism that mitigates oxidative stress and ferroptosis (Zhang et al., 2025). In addition, anaerobic microorganisms *Bacteroides acidifaciens* exhibits protective effects against alcoholic liver disease, colitis, and obesity (Zheng et al., 2023; Shen et al., 2024). Especially, metabolites derived from microbiota are recognized as essential regulators of various diseases. *A. muciniphila* is associated with disease regulation through its metabolites. However, the injection of *A. muciniphila* reinstated levels of proinflammatory cytokines, endotoxins (LPS and LBP), serotonin-related cognitive function, and liver damage. This indicates that *A. muciniphila* is crucial for regulating the gut-liver-brain axis (**Figure 1**) (Pan et al., 2025; Kang et al., 2024; Wu et al., 2022).

## 6 Discussion

The human gut microbiota plays a crucial role in communication among various organs through the production of bioactive molecules. Through these microbial products, the gut can influence metabolic homeostasis, immune responses, epithelial barrier integrity, and inflammatory processes in distant organs. Among the best-characterized microbiota-derived metabolites are short-chain fatty acids (SCFAs), which can regulate cellular energy metabolism through AMP-activated protein kinase (AMPK), a key sensor of cellular energy status. AMPK signaling contributes to the regulation of autophagy, oxidative stress, mitochondrial function, and inflammatory responses in intestinal and pulmonary tissues. In the setting of severe acute pancreatitis (SAP), activation of AMPK may attenuate NLRP3 inflammasome and NF-κB signaling, thereby reducing oxidative and inflammatory injury in alveolar epithelial and endothelial cells. This provides a potential mechanistic link between microbiota-derived SCFAs and the development of SAP-associated acute lung injury (Wang et al., 2023; Olivier et al., 2022; Ahmad et al., 2021).

In addition to SCFAs, microbiota-regulated lipid metabolism represents another axis through which intestinal dysbiosis shapes distal immune responses. Long-chain fatty acids (LCFAs) generated or modulated by the gut microbiota can act directly on intestinal and systemic immune cells to favor pro-inflammatory T-cell programs. In a psoriasis model, dysbiotic microbiota increased colonic levels of the LCFAs oleic and stearic acid, and administration of these fatty acids was sufficient to increase Th17 differentiation and monocyte-derived dendritic cell infiltration, along with IL-23 production by dendritic cells (Zhao Q et al., 2023). Consistent with this, LCFAs acting in the small intestine have been shown to directly promote Th1/Th17 differentiation and exacerbate CNS autoimmune inflammation, in contrast to the protective, Treg-favoring effects of SCFAs such as propionate (Haghikia et al., 2015). Beyond fatty acids themselves, sphingolipid metabolism is also under microbiota- and cytokine-linked control: loss of IL-10 signaling in macrophages elevates very-long-chain ceramide synthesis, which is required for the heightened inflammatory gene expression characteristic of IL-10 deficiency and intestinal inflammation (York et al., 2024).

**Figure 2:**
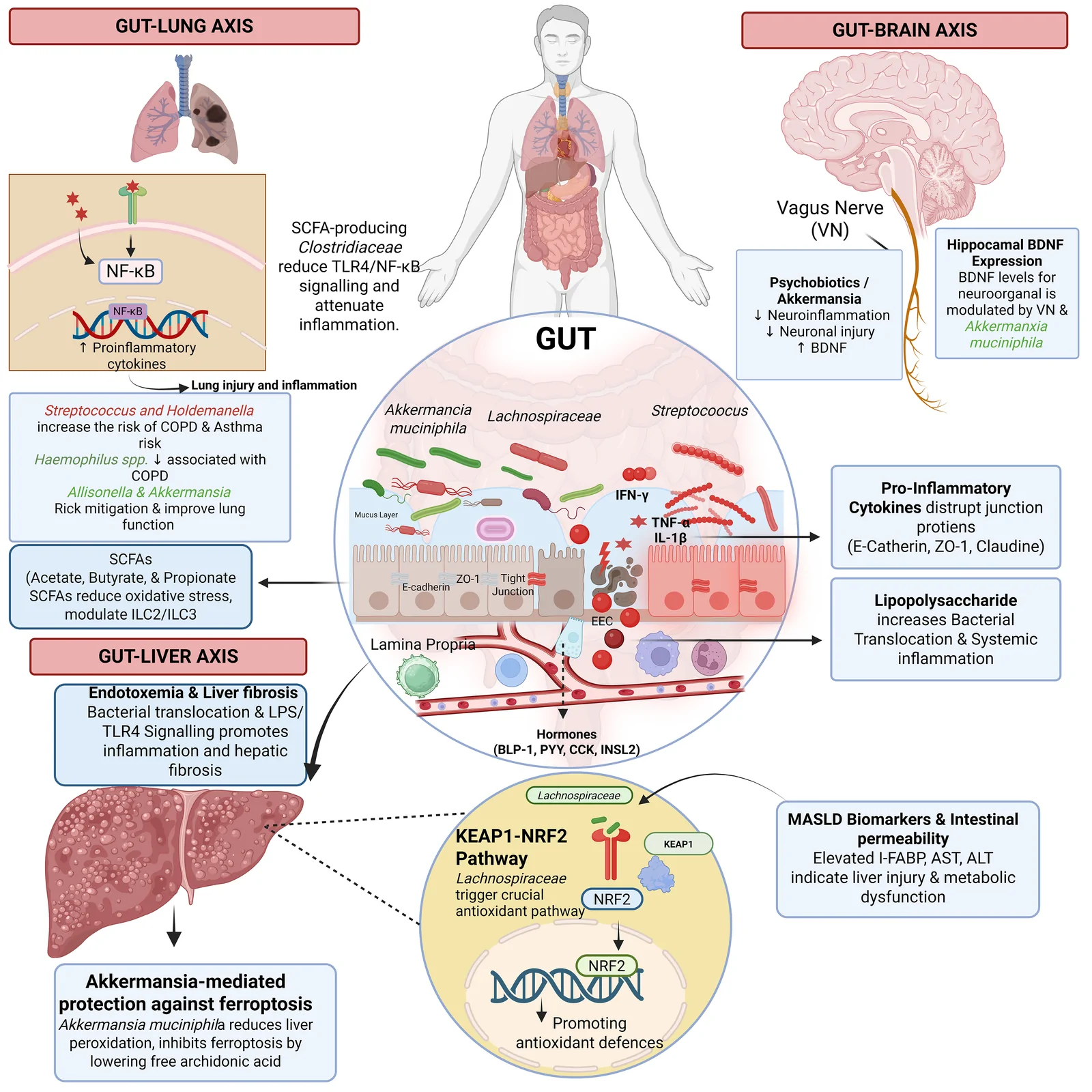
Integrated mechanisms linking gut microbial alterations with gut–lung, gut–brain, and gut–liver outcomes. The figure summarizes how gut microbiota and microbial metabolites influence distant-organ function through immune signalling, epithelial barrier integrity, metabolic pathways, and neuroendocrine communication. The gut–lung axis highlights SCFA-mediated regulation of inflammatory pathways, including TLR4/NF-κB and ILC responses, together with LPS-associated barrier disruption. The gut–brain axis illustrates microbial metabolite and vagal signalling, including potential effects on neuroinflammation, neuronal injury, and BDNF. The gut–liver axis depicts bacterial translocation, TLR4 signalling, oxidative stress, ferroptosis, and potential protective mechanisms involving Lachnospiraceae–KEAP1–NRF2 and *Akkermansia muciniphila*. The central gut compartment integrates the mucus layer, epithelial barrier, immune cells, enteroendocrine cells, and gut-derived hormones. The figure represents a conceptual synthesis of current evidence; microbial associations should not necessarily be interpreted as causal relationships in humans.

Host-related and environmental factors should be carefully considered when interpreting associations between the gut microbiota and pulmonary diseases. Gut microbial composition and respiratory health can both be independently influenced by factors such as smoking history and intensity, age, dietary habits, medication use, biomass smoke exposure, and ambient air pollution. Consequently, differences in gut microbial abundance observed in patients with chronic obstructive pulmonary disease (COPD) and other respiratory disorders may not necessarily reflect disease-specific alterations but may also arise from these underlying lifestyle and environmental factors. Therefore, distinguishing microbiota changes directly associated with respiratory disease from those related to host characteristics or environmental exposures requires well-designed longitudinal studies with comprehensive assessment and appropriate adjustment for potential confounding variables (**Figure 2**) (Liu et al., 2023).

The gut–brain axis represents an important bidirectional communication network through which the gut microbiota can influence neurological, metabolic, and immune functions.Increasing evidence suggests that gut microbiota may contribute to the pathophysiology of Parkinson’s disease (PD) through gut–brain signaling and the microbial metabolism of levodopa. Emerging clinical evidence further suggests that microbiota-directed interventions may influence both gastrointestinal and neurological outcomes. In patients with PD, early interventional studies of fecal microbiota transplantation (FMT) have reported improvements in constipation and selected motor or non-motor symptoms; however, the available evidence remains limited and requires confirmation in larger, well-controlled clinical trials. These findings illustrate the translational potential of microbiota modulation but do not yet establish FMT as a standard treatment for neurological or hepatic disease (Vendrik et al., 2023; DuPont et al., 2023). Beyond neurodegenerative disorders, the gut–liver–brain axis provides another example of microbiota-mediated communication with the central nervous system. In cirrhosis, intestinal dysbiosis and impaired gut barrier function may increase the production or systemic exposure to microbial-derived neuroactive and hepatotoxic metabolites, contributing to hepatic encephalopathy (HE) and associated neurological dysfunction. Therefore, restoration of intestinal microbial and barrier homeostasis may represent a potential therapeutic strategy for reducing gut-derived metabolite exposure and modulating inflammation in cirrhosis and HE, although its clinical efficacy requires further validation (Zhu et al., 2023).

Significantly, there are association exists between the pro-inflammatory factors IL-36γ and IL-38 and the pathogenic bacteria *Veillonella* and *Fusobacterium*. In inflammatory bowel disease (IBD), elevated levels of IL-36γ exacerbate intestinal inflammation and promote wound healing, thereby playing a role in gut inflammatory conditions such as ulcerative colitis (UC) and IBD. IL-38 is elevated in patients with inflammatory bowel disease, contributing to a reduction in intestinal inflammation. IL-38 reduces inflammation by inhibiting IL-36R. This indicates that IL-36γ and IL-38 may serve as markers for liver cirrhosis (LC). The IL-36 subfamily regulates the balance of gut microbiota and inflammation (Fu et al., 2024; Ohno et al., 2022). Hepatic β7+ T cells in primary sclerosing cholangitis associated with inflammatory bowel disease and chronic liver diseases demonstrate elevated production of TNF-α, IFN-γ, and IL-17. The hypothesis posits that hepatic β7+ T cells, derived from the inflammatory gut, exhibit inflammatory traits similar to those of gut lymphocytes (Graham et al., 2022). In addition, the environmental pollutants play a crucial role in multiple disorders including liver damage. Especially, it is correlated with non-alcoholic inflammation in the liver, and the results from the recent research indicated that zebrafish exposed to Microplastics (MPs), oxytetracycline (OTC), and a combination of MP-OTC exhibited elevated Firmicutes/Bacteroidetes ratios alongside a reduction in Proteobacteria compared to the control group. This was demonstrated through an investigation of gut microbiota. MPs and OTC may target the gut-liver axis in MASLD zebrafish. Thus, pollutants such as antibiotics and microplastics may impact patients with MASLD (Zhou et al., 2023).

Kim et al. demonstrated that host defense system against infections is dependent on IL-1β, which is produced by innate immune cells in reaction to pathogens and damage patterns, and gut microorganisms are inherently linked to this process (Kim et al., 2023). Similarly, another study by Breyer et al. found that IL-4 provides protection to the gastrointestinal and respiratory systems. The mouse model for pathogenic *E. coli*, *Citrobacter rodentium*, exhibited improved healing with IL4 treatment, which influenced colitis, mucus production, and pathogen adherence. A recent study demonstrated that stomach ETEC can induce lung IL4 transcription, thereby evading opportunistic infections (Breyer et al., 2025).

Overall, the gut–lung, gut–brain, and gut–liver axes appear to share a common biological framework involving microbial dysbiosis, metabolite signaling, epithelial barrier integrity, and immune regulation, while exhibiting organ-specific downstream responses. The major challenge for the field is therefore to move beyond descriptive microbial associations toward mechanistic and clinically validated models that account for host and environmental variability.

## 7 Conclusion

This comprehensive review analyses the gut microbiota associated with various disorders, particularly those affecting the lungs, brain, and liver. In recent years, several researchers have initiated a relationship between gut microbial communities and different axes. in regulating the gut-brain-liver axis, alongside Proteobacteria and Firmicutes may contribute to the regulation of the gut–brain–liver axis. Thus, understanding gut microbial composition and function may provide insights into the mechanisms linking intestinal dysbiosis with host organ dysfunction. From a clinical perspective, microbiota profiling and measurement of microbiota-derived metabolites may have potential as adjunctive tools for disease stratification, risk assessment, and identification of patients who may benefit from microbiome-targeted interventions. However, differences in microbiota composition between individuals, arising from age, diet, lifestyle, medication use, environmental exposure, host genetics, and disease status, may influence microbiota–disease associations. Therefore, microbial signatures identified in one population may not necessarily have the same diagnostic or therapeutic value in another population, and prospective validation in diverse populations is required before routine clinical implementation.

Future research should move beyond taxonomic profiling toward integrated multi-omics approaches, combining metagenomics, meta-transcriptomics, metabolomics, proteomics, and host genomic information to determine how microbial alterations translate into functional changes in the host. Machine-learning approaches may further assist in integrating complex microbiome, metabolite, and clinical datasets to identify reproducible microbial signatures and improve disease prediction. Longitudinal and intervention-based studies are also needed to distinguish causal microbial alterations from disease consequences and to determine whether microbiome-targeted interventions produce consistent clinical benefits. Collectively, integrating microbiota composition, microbial metabolites, host characteristics, and clinical phenotypes may support a more personalized understanding of microbiota-associated diseases and facilitate the development of clinically useful microbiome-based biomarkers and therapeutic strategies.
